# Temozolomide post pazopanib treatment failure in patients with advanced sarcoma: A case series

**DOI:** 10.1371/journal.pone.0188116

**Published:** 2017-11-15

**Authors:** Manojkumar Bupathi, John L. Hays, James L. Chen

**Affiliations:** 1 Department of Internal Medicine, Division of Medical Oncology, Ohio State University, Columbus, Ohio, United States of America; 2 Department of Obstetrics and Gynecology, Division of Gynecologic Oncology, Ohio State University, Columbus, Ohio, United States of America; 3 Department of Biomedical Informatics, Division of Bioinformatics, Ohio State University, Columbus, Ohio, United States of America; Yeshiva University Albert Einstein College of Medicine, UNITED STATES

## Abstract

**Background:**

Sarcomas are rare, heterogeneous tumors for which prognosis remains dismal in patients with advanced disease. Pazopanib, a vascular endothelial growth factor receptor inhibitor, has shown modest efficacy in patients with soft tissue sarcoma who fail cytotoxic chemotherapy. The cytotoxic agent temozolomide has also demonstrated activity in patients with advanced sarcoma.

**Objective:**

We performed a retrospective case series to evaluate the feasibility of adding temozolomide to pazopanib in advanced sarcoma patients following single-agent pazopanib failure.

**Patients and methods:**

Patients with recurrent, metastatic sarcomas who had progressed on single-agent pazopanib and continued on pazopanib with the addition of temozolomide were included in this retrospective analysis to examine the tolerability and responses associated with the treatment combination.

**Results:**

Nine patients with a range of sarcoma subtypes were identified (55% female; median age, 48 years; median number of therapies prior to pazopanib, 3). All patients received combination therapy. One patient was recently started on therapy and was excluded from the analysis (n = 8 evaluable patients). Median PFS for single-agent pazopanib was 7.5 months (range 2–19). For the eight evaluable patients (63% female), best response at 4 months with pazopanib plus temozolomide was partial response (n = 1), stable disease (n = 3) and progressive disease (n = 4), with a median PFS of 3.5 months (range 0–15). Median PFS with combination treatment in patients with stable disease or response was 8 months (range 5–15). All four patients who achieved clinical benefit remain on therapy and are tolerating the combination therapy with expected but manageable side effects.

**Conclusions:**

In heavily pretreated patients with advanced sarcoma, the addition of temozolomide to pazopanib was found to be tolerable. Future prospective trials are required to deduce whether temozolomide extends the clinical benefit of pazopanib.

## Introduction

Sarcomas are a rare and heterogeneous set of diseases that have a mesenchymal origin in bone or soft tissue. Soft tissue sarcomas (STS) are composed of more than 70 different subtypes and account for approximately 1% of all tumors [1,2]. In 2017 it is estimated that 12,390 people in United States will be diagnosed with STS [3]. The heterogeneity of STS poses a therapeutic challenge. The prognosis for metastatic STS is dismal [4], with a median overall survival (OS) of approximately 12 months [1,5,6] indicating a strong need for new therapeutic options.

Treatment of metastatic disease remains unsatisfactory due to limited active chemotherapy options [1]. Traditionally, first-line treatment for metastatic STS is an anthracycline as monotherapy or in combination with ifosfamide [7]. At the time of progression, other treatment options include gemcitabine in combination with dacarbazine [8], gemcitabine in combination with docetaxel [9,10], or single-agent paclitaxel for angiosarcoma [4,11]. However, combination chemotherapies have historically not been shown to improve OS when compared with single agents [12,13]. Extraskeletal myxoid chondrosarcoma and dedifferentiated chondrosarcoma are aggressive sarcomas that are typically weakly active to cytotoxic chemotherapies [14,15]; recently preliminary evidence of efficacy has been demonstrated with targeted therapies in retrospective studies [16,17]. In 2016 olaratumab, a human platelet-derived growth factor receptor α antibody (anti-PDGFR-α), received US Food and Drug Administration approval for the treatment of STS in combination with doxorubicin [18]. The combination resulted in an impressive OS benefit of 26.5 months in the olaratumab with doxorubicin arm versus 14.7 months in the doxorubicin arm in their registration study [19]. Other sarcoma regimens combining cytotoxic and targeted therapies may be promising.

Pazopanib is a multi-targeted tyrosine kinase inhibitor (TKI) that inhibits vascular endothelial growth factor receptor (VEGFR)-1, -2, and -3; PDGFR-α and -β, and stem cell factor receptor (c-Kit), B-Raf and others [20,21]. In the Phase 3 PALETTE study in patients with progressive, metastatic STS, those treated with pazopanib demonstrated significantly longer median progression-free survival (PFS) compared with those in the placebo group (4.6 months versus 1.6 months) [5]. Importantly, in preclinical and translational studies, pazopanib inhibited activation of both phosphoinositide 3-kinase (PI3K) and MAPK/extracellular signal-regulated kinase (ERK) pathways [21,22], along with the above-mentioned oncogenic pathways in multiple tumor types [20]. Thus, if a cancer cell relies on the activation of PI3K and MAPK pathways for its survival, pazopanib may have enhanced anti-tumor efficacy.

To this end, temozolomide is a novel alkylating agent that exerts its effects through the formation of 5-3-methyl-1-triazenolimidazole-4 carboxamide, which is the putative active metabolite of dacarbazine [23]. Temozolomide, alone or in combination with other forms of cytotoxic chemotherapy, has been demonstrated to have activity in sarcoma [4]. A phase II trial evaluated the efficacy of temozolomide in 25 patients with unresectable or metastatic STS and showed modest activity in terms of PFS and OS: after a median follow-up of 13.2 months, median PFS was 2 months and median OS was 13.2 months [23]. Although the exact mechanism by which temozolomide causes cell death is largely unknown, recent pre-clinical data suggest that there is increased apoptotic activity in multiple sarcoma cell lines treated with temozolomide and altered signaling through the PI3K/Akt pathway and the ERK 1/2 pathway [24]. The authors suggest that increased signaling through these mitogenic pathways may explain the varied response to temozolomide across multiple cell lines [24].

**Fig 1:**
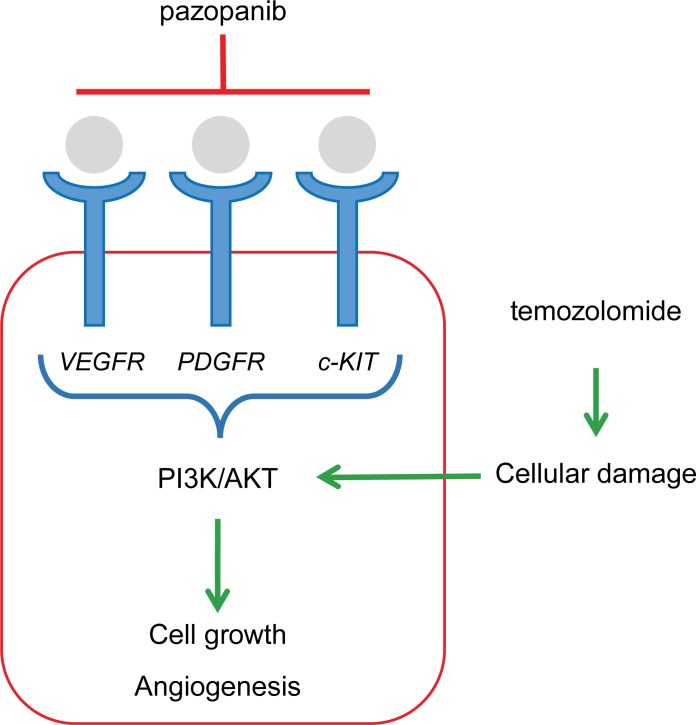
A suggested mechanism for combination therapy with temozolomide and pazopanib. Temozolomide increases cellular damage, necessitating the upregulation of the PI3K/AKT pathway. Pazopanib prevents the upregulation of this signaling, thereby preventing the tumor cell from overcoming the induced damage. Abbreviations: c-KIT = stem cell factor receptor; FGF = fibroblast growth factor; HIF1-α; hypoxia-inducible factor 1-α; PDGF = platelet-derived growth factor; VEGF = vascular endothelial growth factor.

We thus hypothesize that during progression on pazopanib, the tumor cells have begun developing escape pathways around the tyrosine kinase inhibition. However, the addition of temozolomide after progression on pazopanib may re-induce a tumor response by inducing cellular damage that necessitates the upregulation of the PI3K/Akt pathway. Despite the upregulation of escape pathways, the tumor now becomes increasingly dependent on PI3K/Akt activation; however, this activation remains downregulated by pazopanib (Fig 1), leading to a clinical response. Bevacizumab, a specific anti-VEGF therapy has activity in STS [4,25]. Pre-clinical models have shown that anti-VEGF therapy leads to increased tumor invasion and migration when given without cytotoxic chemotherapy [26,27]. Continued use of anti-VEGF could result in tumor escape through vascular co-option [26,28], which ultimately could lead to disease progression due to intratumoral signaling. This can be seen more commonly in highly vascularized tumors such as glioblastomas multiforme [26]. The use of cytotoxic chemotherapy can lead to cellular damage, leading to increased repair enzymes, and ultimately leading to increased levels of VEGF, which is then inhibited by pazopanib. Grossman et al. evaluated the effect of combination of anti-VEGF therapy with temozolomide in an experimental malignant glioma model and showed that combination therapy does not reduce the efficacy of either drug and can significantly improve median survival [29]. Furthermore, in glioma cell lines there is a suggestion that cediranib, a highly potent VEGFR inhibitor (VEGFRi), enhanced the effectiveness of temozolomide [30]. In this case series, we evaluated the feasibility of adding temozolomide to pazopanib in patients with advanced sarcoma who had progressed on pazopanib.

## Methods

### Study design and patient selection

We conducted a retrospective analysis of data from patients with advanced sarcoma who received pazopanib plus temozolomide at our institution from January 1, 2014 to June 30, 2016, and who were evaluable for toxicity and tumor response. The study was approved by the Ohio State University Institutional Review Board (OSU: 2016C0022).

Patients were eligible for inclusion in the study if they were older than 18 years of age with a pathologically-confirmed diagnosis of soft tissue or bone sarcoma, had received therapy with a single-agent VEGFRi (pazopanib) in the recurrent or metastatic setting and were subsequently treated with pazopanib in combination with temozolomide. Patients were required to have received at least one cycle of pazopanib-based therapy prior to combination with temozolomide.

Data collection (age, sex, histology, prior treatments, stage at diagnosis, toxicity, response, and survival) was performed by a thorough retrospective review of each patient’s medical record.

### Adverse event evaluation

Adverse events were evaluated using Common Terminology Criteria for Adverse Events version 4.0.

### Efficacy evaluation

PFS, defined as the time from the date of clinic visit prior to starting temozolomide plus pazopanib to disease progression or death, whichever occurred first. Patients were censored if there was no progression at the time of last follow up. All patients were evaluated with computed tomography scans every 8 weeks. Response to therapy was defined according to RECIST version 1.1 criteria and evaluated by the treating physician.

### Statistical analysis

Demographics, patient characteristics, toxicities, and clinical response were summarized using descriptive statistics (median/range for continuous outcomes, and proportions for categorical outcomes). Survival curves were estimated using the Kaplan-Meier method.

## Results

### Patient characteristics

Nine patients were identified and eight patients with a range of sarcoma subtypes were evaluated in this analysis (Table 1). At the time of analysis one patient had started on combination therapy 1 month prior, completed one cycle and was tolerating therapy well. This patient was excluded from the analysis since no response data was yet available. Five patients (63%) were female and median age was 48 years (range 21–69). Treatment received prior to pazopanib is shown in Table 1, with a median number of prior therapies of 3 (range 1–4).

**Table 1:** Sarcoma subtype, prior therapy, duration of treatment and best response with pazopanib monotherapy.

Patient ID	Sarcoma subtype	Prior therapy	Duration of treatment with pazopanib monotherapy (months)	Best response to pazopanib monotherapy
1	Sarcoma NOS	Surgery	8	SD
2	Extraskeletal myxoid chondrosarcoma	Sunitinib, radiation, nivolumab	19	SD
3	Non-uterine leiomyosarcoma	AIM, radiation, denosumab, gemcitabine/docetaxel	4	SD
4	Dedifferentiated chondrosarcoma	Everolimus	15	SD
5	Non-uterine leiomyosarcoma	Gemcitabine/docetaxel ± ontuxizumab, doxorubicin, received single-agent bevacizumab	7	SD
6	Small blue round cell tumor	VAC/IE, radiation with irinotecan	2	PD
7	Epithelioid sarcoma	AIM, radiation with paclitaxel, gemcitabine/docetaxel	2	PD
8	Spindle cell sarcoma	Radiation	8	SD

AIM = doxorubicin, ifosfamide, mesna; NOS = not otherwise specified; PD, progressive disease; SD, stable disease; VAC/IE = vincristine, adriamycin, cyclophosphamide alternating with ifosfamide and etoposide.

### Treatment

**Fig 2:**
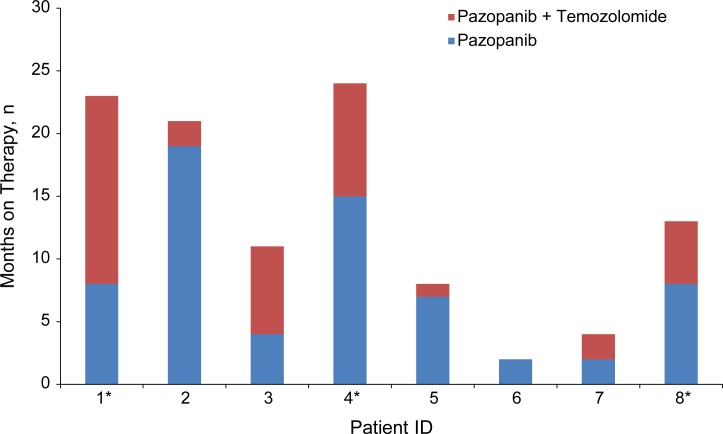
Duration of therapy with single-agent pazopanib followed by pazopanib plus temozolomide. *Patient still on therapy at time of analysis.

All patients received pazopanib (800 mg orally per day) until progression. Median PFS for single-agent pazopanib was 7.5 months (range 2–19) with a best response of stable disease (SD) for most patients (Table 1). At the time of progression, all eight evaluable patients continued on pazopanib (400 mg/day and increased to 800 mg/day if tolerated) daily and also initiated temozolomide (150 mg/m^2^, 7 days on with 7 days off) on a 28-day cycle (Fig 2).

### Adverse events during combination therapy

The combination therapy was tolerated, with only one patient having a grade 4 toxicity of thrombocytopenia (Table 2). Proteinuria was assessed and was not observed.

**Table 2:** Adverse events by grade.

	Grade 1	Grade 2	Grade 3	Grade 4
**Non-hematologic**				
Fatigue	4	3	0	0
Neuropathy	5	0	0	0
Anorexia	4	0	0	0
Nausea	6	1	0	0
Constipation	4	0	0	0
Diarrhea	3	1	0	0
Rash	1	1	0	0
Hypertension	4	2	0	0
Mucositis	0	1	0	0
Depression	3	1	0	0
**Hematologic**				
Neutropenia	1	0	0	0
Thrombocytopenia	0	2	1	1

### Response to pazopanib plus temozolomide following pazopanib failure

The combination of pazopanib plus temozolomide resulted in disease stabilization as best response in three of eight patients at 4 months (Table 3). The clinical benefit rate (partial response [PR] + complete response [CR] + SD]) based on best response to combination therapy at 4 months was 50% (4/8). Best response over 4 months was PR (n = 1), SD (n = 3) and progressive disease (PD) (n = 4). Median PFS was 3.5 months (range 0–15). One patient with PD as best response discontinued treatment due to rapidly progressive disease and did not receive one complete cycle of the combination therapy as they were too ill. The median PFS was 8 months (range 5–15) in patients who achieved some clinical benefit (PR/SD). At the time of analysis, three of four patients were still on therapy and tolerating the combination well.

**Table 3:** Best response to pazopanib plus temozolomide at 4 months.

Patient ID	Sarcoma subtype	Best response to pazopanib plus temozolomide at 4 months	Duration of treatment with pazopanib plus temozolomide (months)
1*	Sarcoma NOS	PR	15
2	Extraskeletal myxoid chondrosarcoma	PD	2
3	Non-uterine leiomyosarcoma	SD	7
4*	Dedifferentiated chondrosarcoma	SD	9
5	Non-uterine leiomyosarcoma	PD	1
6	Small blue round cell tumor	PD	0
7	Epithelioid sarcoma	PD	2
8*	Spindle cell sarcoma	SD	5

*Patient still on therapy at time of analysis.

NOS = not otherwise specified; PD = progressive disease; PR = partial response; SD = stable disease.

### Genomic alterations

In addition, we also evaluated next-generation sequencing for all patients to identify if there are any key genomic alternations that could be used as a possible predictive biomarker. We did not find any similarities among the patients (Table 4).

**Table 4:** Genomic alterations.

	Alterations
**Patient 1**	CDK4 (amp), ERBB3 (amp), MDM2 (amp), FRS2 (amp)
**Patient 2**	EGFR (splice site 2061+2T>C), EWSR1 fusion (EWSR1-NR4A3)
**Patient 3**	TP53(Q192)
**Patient 4**	Not Available
**Patient 5**	TP53(I162F), TSC2(A607T)
**Patient 6**	CDC73, CIC, FANCC
**Patient 7**	SMARCB1
**Patient 8**	CTNNA1 (R731), TP53(C176F)

Amp = amplification.

## Discussion

Metastatic STS are difficult to treat and typically resistant to cytotoxic chemotherapy, with a poor overall prognosis [1,4]. With the use of next-generation sequencing, novel aberrations have been identified and the use of targeted therapies has been beneficial in specific subsets of STS patients, albeit with short-lived activity [4]. Preclinical data suggests that temozolomide has activity in sarcoma cell lines, specifically altering the effect of PI3K/Akt pathway [24]. Inhibition of VEGFR and other tyrosine kinase receptors with pazopanib has been shown to improve PFS relative to placebo in patients with STS [5] and also alter signaling through PI3K/Akt and MAPK pathways [21,22].

This is the first case series, to our knowledge, evaluating the feasibility of the combination of pazopanib and temozolomide in patients with advanced sarcoma. Our data, although retrospective, comprising a small sample size, and a range of sarcoma subtypes, tentatively suggest that this combination is tolerable and that further investigation of the efficacy of this regimen in a subset of patients with refractory sarcoma may be worth consideration. The ability of this combination regimen to help overcome tolerance to single-agent pazopanib cannot be deduced from this case series and would need to be assessed in a prospective randomized clinical trial vs a group receiving temozolomide monotherapy. At our institution, the starting dose of pazopanib (400 mg/day) administered in combination with temozolomide was lower than the approved dose of 800 mg/day [20] and was selected based on studies demonstrating a requirement for significant dose modifications of VEGFRi (vatalanib and vandetanib) when combined with temozolomide. [31,32] The intermittent dosing of temozolomide used at our institution (150 mg/m^2^ for 7 days on, 7 days off) was selected based on previous studies using the combination of temozolomide and the VEGFi bevacizumab in the treatment of solitary fibrous tumors [33].

The median PFS for patients receiving combination treatment in the present study was 3.5 months with a range from 0–15. There were three patients in our cohort who were started on combination therapy and who were already experiencing rapid progression of disease to the point of requiring an ICU admission. The median PFS for these three patients was 1 month compared to 8 months for the remainder of the patients. This suggests that there could be two distinct populations of patients. One that achieves no benefit from the combination therapy and is generally not likely to tolerate even one cycle, and a second population that may achieve slight benefit from the combination, but where potentially neither population may be considered a true responder to combination therapy.

In this population, patients had a median time of 7.5 months (range 2–19) on pazopanib monotherapy. The median time on pazopanib monotherapy in this case series appears to be longer than that in the PALETTE trial, wherein the median duration of pazopanib treatment was 16.4 weeks (range 0–79) [5]. This is likely reflective of the low number of patients and the wide range of sarcoma subtypes evaluated in this case series vs the PALETTE trial which randomized 369 patients and only included the most common histological subtypes of STS. Also, the PALETTE trial included patients who had progressed on at least one prior standard chemotherapy. The addition of temozolomide in this study resulted in clinical benefit in four of eight patients, with three patients remaining on combination therapy. In comparison, phase II studies in patients with refractory or metastatic STS demonstrated a clinical benefit rate with temozolomide monotherapy of between 20% and 34% [23,34,35]. The PFS that was reported in these studies was approximately 2 months [23,34,35], and patients with leiomyosarcoma had the greatest benefit to response [23,34]. Interestingly, although our cohort contained two patients with leiomyosarcoma, these are not the patients who derived the greatest degree of clinical benefit. Although the findings here tentatively indicate the potential benefit of combination therapy in these patients, no firm conclusions can be drawn since direct comparisons cannot be made between the studies. Accordingly, these findings would be interesting to explore further in the context of a prospective study.

Limitations of this study include the retrospective nature, range of sarcomas included, and small sample size. Adverse event data may not have been collected in a uniform fashion compared to those participating in a clinical trial. Next-generation sequencing performed on these patients as part of routine clinical practice failed to identify any key genomic alterations that could be used as a possible predictive biomarker.

In conclusion, our retrospective case series evaluated the feasibility of the addition of temozolomide to pazopanib in heavily pretreated patients with advanced sarcoma, who had predominantly begun developing slow disease progression. The combination of temozolomide with pazopanib was found to be tolerable. In order to determine whether temozolomide extends the clinical benefit of pazopanib vs temozolomide monotherapy, a large, randomized, prospective study would need to be completed. Next generation sequencing could aid in the identification of sarcoma subtype-specific alterations and help elucidate key predictive biomarkers.

## References

[1] Clark MA, Fisher C, Judson I, Thomas JM. Soft-tissue sarcomas in adults. N Engl J Med. 2005; 353(7): 701–711. doi: 10.1056/NEJMra041866 16107623 10.1056/NEJMra041866

[2] Pang A, Carbini M, Maki RG. Contemporary therapy for advanced soft-tissue sarcomas in adults: a review. JAMA Oncol. 2016; 2(7): 941–947. doi: 10.1001/jamaoncol.2016.0241 27148906 10.1001/jamaoncol.2016.0241

[3] American Cancer Society, Inc. Cancer Facts and Figures 2017. Available from: https://www.cancer.org/content/dam/cancer-org/research/cancer-facts-and-statistics/annual-cancer-facts-and-figures/2017/cancer-facts-and-figures-2017.pdf. Accessed January 26, 2017.

[4] National Comprehensive Cancer Network,Inc. NCCN Clinical Practice Guidelines in Oncology. Soft Tissue Sarcoma. Version 1.2017. Published December 21, 2016.

[5] van der Graaf WT, Blay JY, Chawla SP, Kim DW, Bui-Nguyen B, Casali PG, et al Pazopanib for metastatic soft-tissue sarcoma (PALETTE): a randomised, double-blind, placebo-controlled phase 3 trial. Lancet. 2012; 379: 1879–1886. doi: 10.1016/S0140-6736(12)60651-5 22595799 10.1016/S0140-6736(12)60651-5

[6] Sleijfer S, Ouali M, Van GM, Krarup-Hansen A, Rodenhuis S, Le CA, et al Prognostic and predictive factors for outcome to first-line ifosfamide-containing chemotherapy for adult patients with advanced soft tissue sarcomas: an exploratory, retrospective analysis on large series from the European Organization for Research and Treatment of Cancer-Soft Tissue and Bone Sarcoma Group (EORTC-STBSG). Eur J Cancer. 2010; 46(1): 72–83. doi: 10.1016/j.ejca.2009.09.022 19853437 10.1016/j.ejca.2009.09.022

[7] Leahy M, Garcia del Muro X, Reichardt P, Judson I, Staddon A, Verweij J, et al Chemotherapy treatment patterns and clinical outcomes in patients with metastatic soft tissue sarcoma. The SArcoma treatment and Burden of Illness in North America and Europe (SABINE) study. Ann Oncol. 2012; 23(10): 2763–2770. doi: 10.1093/annonc/mds070 22492696 10.1093/annonc/mds070

[8] Garcia del Muro X, Lopez-Pousa A, Maurel J, Martin J, Martinez-Trufero J, Casado A, et al Randomized phase II study comparing gemcitabine plus dacarbazine versus dacarbazine alone in patients with previously treated soft tissue sarcoma: a Spanish Group for Research on Sarcomas study. J Clin Oncol. 2011; 29(18): 2528–2533. doi: 10.1200/JCO.2010.33.6107 21606430 10.1200/JCO.2010.33.6107

[9] Maki RG. Gemcitabine and docetaxel in metastatic sarcoma: past, present, and future. Oncologist. 2007; 12(8): 999–1006. doi: 10.1634/theoncologist.12-8-999 17766660 10.1634/theoncologist.12-8-999

[10] Maki RG, Wathen JK, Patel SR, Priebat DA, Okuno SH, Samuels B, et al Randomized phase II study of gemcitabine and docetaxel compared with gemcitabine alone in patients with metastatic soft tissue sarcomas: results of sarcoma alliance for research through collaboration study 002 [corrected]. J Clin Oncol. 2007; 25(19): 2755–2763. doi: 10.1200/JCO.2006.10.4117 17602081 10.1200/JCO.2006.10.4117

[11] Penel N, Bui BN, Bay JO, Cupissol D, Ray-Coquard I, Piperno-Neumann S, et al Phase II trial of weekly paclitaxel for unresectable angiosarcoma: the ANGIOTAX Study. J Clin Oncol. 2008; 26(32): 5269–5274. doi: 10.1200/JCO.2008.17.3146 18809609 10.1200/JCO.2008.17.3146

[12] Antman K, Crowley J, Balcerzak SP, Rivkin SE, Weiss GR, Elias A, et al An intergroup phase III randomized study of doxorubicin and dacarbazine with or without ifosfamide and mesna in advanced soft tissue and bone sarcomas. J Clin Oncol. 1993; 11(7): 1276–1285. doi: 10.1200/JCO.1993.11.7.1276 8315425 10.1200/JCO.1993.11.7.1276

[13] Edmonson JH, Ryan LM, Blum RH, Brooks JS, Shiraki M, Frytak S, et al Randomized comparison of doxorubicin alone versus ifosfamide plus doxorubicin or mitomycin, doxorubicin, and cisplatin against advanced soft tissue sarcomas. J Clin Oncol. 1993; 11(7): 1269–1275. doi: 10.1200/JCO.1993.11.7.1269 8315424 10.1200/JCO.1993.11.7.1269

[14] Stacchiotti S, Dagrada GP, Sanfilippo R, Negri T, Vittimberga I, Ferrari S, et al Anthracycline-based chemotherapy in extraskeletal myxoid chondrosarcoma: a retrospective study. Clin Sarcoma Res. 2013; 3(1): 16 doi: 10.1186/2045-3329-3-16 24345066 10.1186/2045-3329-3-16 PMC3879193

[15] Italiano A, Mir O, Cioffi A, Palmerini E, Piperno-Neumann S, Perrin C, et al Advanced chondrosarcomas: role of chemotherapy and survival. Ann Oncol. 2013; 24(11): 2916–2922. doi: 10.1093/annonc/mdt374 24099780 10.1093/annonc/mdt374 PMC3811906

[16] Stacchiotti S, Pantaleo MA, Astolfi A, Dagrada GP, Negri T, Dei Tos AP, et al Activity of sunitinib in extraskeletal myxoid chondrosarcoma. Eur J Cancer. 2014; 50(9): 1657–1664. doi: 10.1016/j.ejca.2014.03.013 24703573 10.1016/j.ejca.2014.03.013

[17] Paoluzzi L, Cacavio A, Ghesani M, Karambelkar A, Rapkiewicz A, Weber J, Rosen G. Response to anti-PD1 therapy with nivolumab in metastatic sarcomas. Clin Sarcoma Res. 2016; 6: 24 doi: 10.1186/s13569-016-0064-0 28042471 10.1186/s13569-016-0064-0 PMC5200964

[18] Lartruvo [prescribing information]. Indianapolis, IN: Eli Lilly & Co; 2017.

[19] Tap WD, Jones RL, Van Tine BA, Chmielowski B, Elias AD, Adkins D, et al Olaratumab and doxorubicin versus doxorubicin alone for treatment of soft-tissue sarcoma: an open-label phase 1b and randomised phase 2 trial. Lancet. 2016; 388: 488–497. doi: 10.1016/S0140-6736(16)30587-6 27291997 10.1016/S0140-6736(16)30587-6 PMC5647653

[20] Votrient [prescribing information]. East Hanover, NJ: Novartis Pharmaceuticals Corp; 2017.

[21] Gril B, Palmieri D, Qian Y, Smart D, Ileva L, Liewehr DJ, et al Pazopanib reveals a role for tumor cell B-Raf in the prevention of HER2+ breast cancer brain metastasis. Clin Cancer Res. 2011; 17: 142–153. doi: 10.1158/1078-0432.CCR-10-1603 21081656 10.1158/1078-0432.CCR-10-1603 PMC3059742

[22] Hosaka S, Horiuchi K, Yoda M, Nakayama R, Tohmonda T, Susa M, et al A novel multi-kinase inhibitor pazopanib suppresses growth of synovial sarcoma cells through inhibition of the PI3K-AKT pathway. J Orthop Res. 2012; 30(9): 1493–1498. doi: 10.1002/jor.22091 22359392 10.1002/jor.22091

[23] Talbot SM, Keohan ML, Hesdorffer M, Orrico R, Bagiella E, Troxel AB, et al A phase II trial of temozolomide in patients with unresectable or metastatic soft tissue sarcoma. Cancer. 2003; 98: 1942–1946. doi: 10.1002/cncr.11730 14584078 10.1002/cncr.11730

[24] Kusabe Y, Kawashima H, Ogose A, Sasaki T, Ariizumi T, Hotta T, et al Effect of temozolomide on the viability of musculoskeletal sarcoma cells. Oncol Lett. 2015; 10(4): 2511–2518. doi: 10.3892/ol.2015.3506 26622881 10.3892/ol.2015.3506 PMC4580084

[25] Avastin [prescribing information]. South San Francisco, CA: Genentech, Inc; 2016.

[26] de Groot JF, Fuller G, Kumar AJ, Piao Y, Eterovic K, Ji Y, et al Tumor invasion after treatment of glioblastoma with bevacizumab: radiographic and pathologic correlation in humans and mice. Neuro Oncol. 2010; 12: 233–242. doi: 10.1093/neuonc/nop027 20167811 10.1093/neuonc/nop027 PMC2940588

[27] Du R, Lu KV, Petritsch C, Liu P, Ganss R, Passegue E, et al HIF1alpha induces the recruitment of bone marrow-derived vascular modulatory cells to regulate tumor angiogenesis and invasion. Cancer Cell. 2008; 13: 206–220. doi: 10.1016/j.ccr.2008.01.034 18328425 10.1016/j.ccr.2008.01.034 PMC2643426

[28] Donnem T, Hu J, Ferguson M, Adighibe O, Snell C, Harris AL, et al Vessel co-option in primary human tumors and metastases: an obstacle to effective anti-angiogenic treatment? Cancer Med. 2013; 2(4): 427–436. doi: 10.1002/cam4.105 24156015 10.1002/cam4.105 PMC3799277

[29] Grossman R, Brastianos H, Blakeley JO, Mangraviti A, Lal B, Zadnik P, et al Combination of anti-VEGF therapy and temozolomide in two experimental human glioma models. J Neurooncol. 2014; 116(1): 59–65. doi: 10.1007/s11060-013-1268-2 24185441 10.1007/s11060-013-1268-2 PMC4037922

[30] Wachsberger PR, Lawrence RY, Liu Y, Xia X, Andersen B, Dicker AP. Cediranib enhances control of wild type EGFR and EGFRvIII-expressing gliomas through potentiating temozolomide, but not through radiosensitization: implications for the clinic. J Neurooncol. 2011; 105(2): 181–190. doi: 10.1007/s11060-011-0580-y 21516367 10.1007/s11060-011-0580-y

[31] Gerstner ER, Eichler AF, Plotkin SR, Drappatz J, Doyle CL, Xu L, et al Phase I trial with biomarker studies of vatalanib (PTK787) in patients with newly diagnosed glioblastoma treated with enzyme inducing anti-epileptic drugs and standard radiation and temozolomide. J Neurooncol. 2011; 103(2): 325–332. doi: 10.1007/s11060-010-0390-7 20821342 10.1007/s11060-010-0390-7 PMC4090923

[32] Drappatz J, Norden AD, Wong ET, Doherty LM, Lafrankie DC, Ciampa A, et al Phase I study of vandetanib with radiotherapy and temozolomide for newly diagnosed glioblastoma. Int J Radiat Oncol Biol Phys. 2010; 78(1): 85–90. doi: 10.1016/j.ijrobp.2009.07.1741 20137866 10.1016/j.ijrobp.2009.07.1741

[33] Park MS, Patel SR, Ludwig JA, Trent JC, Conrad CA, Lazar AJ, et al Activity of temozolomide and bevacizumab in the treatment of locally advanced, recurrent, and metastatic hemangiopericytoma and malignant solitary fibrous tumor. Cancer. 2011; 117(21): 4939–4947. doi: 10.1002/cncr.26098 21480200 10.1002/cncr.26098 PMC3135685

[34] Garcia del Muro X, Lopez-Pousa A, Martin J, Buesa JM, Martinez-Trufero J, Casado A, et al A phase II trial of temozolomide as a 6-week, continuous, oral schedule in patients with advanced soft tissue sarcoma: a study by the Spanish Group for Research on Sarcomas. Cancer. 2005; 104(8): 1706–1712. doi: 10.1002/cncr.21384 16134177 10.1002/cncr.21384

[35] Woll PJ, Judson I, Lee SM, Rodenhuis S, Nielsen OS, Buesa JM, et al Temozolomide in adult patients with advanced soft tissue sarcoma: a phase II study of the EORTC Soft Tissue and Bone Sarcoma Group. Eur J Cancer. 1999; 35(3): 410–412. 10448291 10.1016/s0959-8049(98)00403-1

